# Leaf transcriptome data of two tropical medicinal plants: *Sterculia lanceolata* and *Clausena excavata*

**DOI:** 10.1016/j.dib.2019.104297

**Published:** 2019-08-02

**Authors:** Seok Hyun Eom, Jong-Kuk Na

**Affiliations:** a Department of Horticultural Biotechnology, Kyung Hee University, Yongin, 17104, Republic of Korea; b Department of Controlled Agriculture, Kangwon National University, Chuncheon, Kangwon, 24341, Republic of Korea

**Keywords:** Transcriptome analysis, Medicinal plant, *Sterculia lanceolata*, *Clausena excavata*

## Abstract

The data presented in this article are associated to the research articles, “DOI: 10.1007/s11295-019-1348-3”, [1]; and “DOI: 10.1007/s13205-018-1162-x” [2]. *Clausena excavata* Burm. f. and *Sterculia lanceolata* Cav. are medicinal tree plants [3,4] native to Southeast Asia and China, and most members of both the genus *Clausena* and the genus *Sterculia* contain various valuable secondary metabolites with a great potential for drug development. Though many phytochemical studies have been conducted using plant extracts from various parts of these plants [4,5], there are very limited genetic resources available. RNA sequencing of *C. excavata* and *S. lanceolata* was conducted using pair-end Illumina HiSeq2500 sequencing system, from which the first *de novo* transcriptome data were produced for both genus *Clausena* and *Sterculia*. Transcriptome shotgun assembly using three different assembly tools [2] generated a total of 16,638 non-redundant contigs (N50, 900 bp) from *C. excavata* and 7,857 (N50, 423 bp) from *S. lanceolata*. The data are accessible at NCBI BioProject: PRJNA428402 for *C. excavata* [2] or PRJNA435648 for *S. lanceolata*[1].

**Table:** Specifications Table

Subject area	Plant Science
More specific subject area	Transcriptomics
Type of data	Table, figure, text file
How data was acquired	RNA sequence data obtained from RNA sequencing using Illumina HiSeq 2500 sequencing platform
Data format	Raw, analyzed
Experimental factors	Total RNAs were isolated from the leaves.
Experimental features	*De novo* transcriptome assembly and analysis were conducted. Leaf samples of both *Sterculia lanceolata* and *Clausena excavata* were collected in the wild because they are not cultivated agriculturally.
Data source location	*Clausena excavata*: Vinh Phuc province, Vietnam (N 16° 26′, E 106° 58′) *Sterculia lanceolata*: Ha Noi, Vietnam (N 14° 50′, E 108° 41′)
Data accessibility	https://www.ncbi.nlm.nih.gov/bioproject/?term=PRJNA428402https://www.ncbi.nlm.nih.gov/bioproject/?term=PRJNA435648

**Table:** 

**Value of the data** • These data are the first *de novo* leaf transcriptome from the genus *Sterculia* and the genus *Clausena*, which increased significantly not only the amount of sequence information available to both genus but also a potential for the discovery of genes involved in biosynthesis of useful secondary metabolites in both species. • The data would be very useful for genetic and comparative studies of *Clausena* or *Sterculia* species as well as their relative species. • Assembled sequences will serve as a reference for future studies and would be valuable resources to examine molecular characteristics involved in pharmaceutical properties of *Sterculia* and *Clausena* species.

## 1 Data

**Fig. 1:**
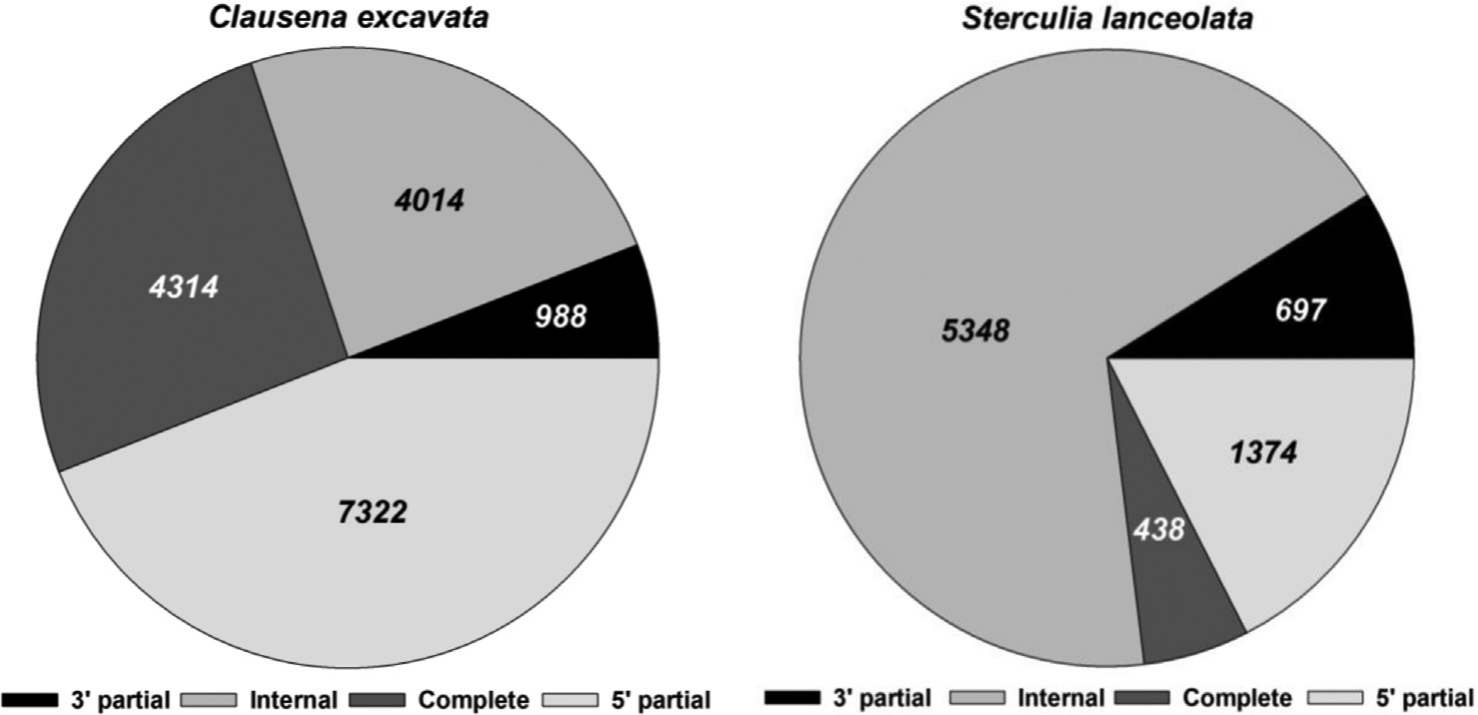
Summary of coding sequences of the contigs from transcriptome data of *C. excavata* and *S. lanceolata*. Contigs with the “5′ partial” only contain start codon in open reading frame, whereas contigs with “3′ partial” contain stop codon in open reading frame. Contigs designated to “internal” do not have both start and stop codon. Contigs with “complete” contain both start and stop codon in open reading frame.

This article reports RNA sequencing transcriptome data from leaf samples of two medicinal plants, *C. excavata* and *S. lanceolata* [3], [4], [5]. The raw read data were deposited at NCBI Sequence Read Archive (SRA) database under the accession SRR6438389 for *C. excavata* [2] and SRR6798190 for *S. lanceolata* [1]. Assembled sequence data are accessible at Transcriptome Shotgun Assembly (TSA) under the accession GEM00000000 for *C. excavata* [2] and GGIS00000000 for *S. lanceolata* [1]. The annotation of the assembled contigs showed that many contigs contain only partial coding regions as shown in Fig. 1. The raw and assembled RNA sequencing data are summarized in Table 1. Simple sequence repeat (SSR) primer sets (464 primer sets from *C. excavata* and 153 sets from *S. lanceolata*), most of which has not been reported and tested, were shown in Supplementary file 1.

**Table 1:** Summary of raw and assembled sequence data.

Description of sequence data	Sequence data	
	*Clausena excavata*	*Sterculia lanceolata*
Number of raw reads	10,348,544	4,357,001
Total length of raw reads (bp)	2,607,833,088	1,097,964,252
Number of filtered clear reads	8,790,228	4,240,923
Total length of filtered reads (bp)	2,143,847,087	1,054,277,267
Percentage of filtered read length (%)	82.2	96.0
Number of assembled contigs	16,638	7,857
GC contents of contigs (%)	43.7	45.7
Shortest and longest contigs (bp)	297 ∼ 4,065	297 ∼ 5,754
Total length of assembled contigs (bp)	12,557,892	3,559,905
Average length (bp)	754.8	453.1
N25 (bp)	1,302	609
N50 (bp)	900	423
N75 (bp)	582	348

## 2 Experimental design, materials and methods

### 2.1 Sample collection

Leaf samples of fully grown wild *C. excavata* Burm. f. and *S. lanceolata* Cav. were collected from Vinh Phuc province or Me Linh field station, Hanoi, Vietnam, August 2015. Leaf samples were submerged into liquid nitrogen, transferred into RNAlater solution (Ambion Ins, USA), and then stored in −20 °C freezer.

### 2.2 cDNA library construction and sequencing

Leaf samples were removed from RNAlater solution and ground with a pestle and mortar in liquid nitrogen to isolate total RNA using TRIzol reagent (Thermo Fisher Scientific, Korea). The purity and quantity of total RNAs were measured using an RNA Pico Chip on the Agilent 2100 Bioanalyzer (Agilent Technologies, USA). A ten μg of the total RNA was used for mRNA isolation using oligo-dT beads, and random sheared mRNA was used for cDNA synthesis, followed by the adaptor ligation at 3’ A overhang. The mRNA isolation and cDNA library construction was conducted by following the procedure of the Sureselect strand-specific RNA reagent kit (Agilent, USA). Equal quantity of mRNA from three different leaf samples from three independent trees was pooled and used for cDNA library construction. The cDNA library was checked for quality using Agilent DNA 1000 chip (Agilent Technologies, USA) and sequenced by the Illumina Hiseq 2500 (Illumina, USA).

### 2.3 *De novo* assembly

The raw reads from sequencing were trimmed and filtered to remove adaptor sequences, empty reads, and low quality reads with ≤20 of a phred quality score and ≤50bp in length using NGS tool kits and Trimmomatic tool [6]. The high quality reads were assembled using three assemblers, CLC Genomics Workbench (ver. 3.7.1), Velvet-Oases (ver. 1.1.04-ver. 0.1.21), and Trinity (release 20110519) with various k-mer lengths. A default k-mer value (25-mer) was used for the assembly with CLC. For the assembly by Velvet-Oases and Trinity, different k-mer values (21–79 for Velvet-Oases; 25 to 33 for Trinity) were applied to obtain the best results. All contigs from each assembler at various k-mer values were merged separately for further process. As Oases does not cluster assembled contigs, CD-HIT-EST was used to cluster the contigs with an identity more than 90% and coverage of 100% [7]. All data sets from each assembler were combined into a single dataset by collapsing identical or near-identical contigs into single contig using CD-HIT-EST with the same criteria described above. Due to the lack of a public reference genome sequence data of both *C. excavata* and *S. lanceolata*, the contigs were annotated by running NCBI BLAST with a cutoff E-value of 10^−6^ against the NCBI non-redundant (NR) protein database.

## Conflict of interest

The authors declare that they have no known competing financial interests or personal relationships that could have appeared to influence the work reported in this paper.

## Appendix A Supplementary data

Simple sequence repeat (SSR) primer sets from transcriptome data from *C. excavata* and *S. lanceolata*.

## References

[1] Eum S.M. (2019). Transcriptome analysis and development of SSR markers of ethnobotanical plant *Sterculia lanceolata*. Tree Genet. Genomes.

[2] Bae D.Y. (2018). Enrichment of genomic resources and identification of simple sequence repeats from medicinally important *Clausena excavata*. 3 Biotech.

[3] Panyaphua K. (2011). Medicinal plants of the Mien (Yao) in Northern Thailand and their potential value. J. Ethnopharmacol..

[4] Arbab I.A. (2012). A review of traditional uses, phytochemical and pharmacological aspects of selected members of *Clausena* genus (Rutaceae). J. Med. Plants Res..

[5] El-Sherei M.M. (2016). Phytochemistry, biological activities and economical uses of the genus *Sterculia* and the related genera. A reveiw Asian Pacific Journal of Tropical Disease.

[6] Bolger A.M., Lohse M., Usadel B. (2014). Trimmomatic: a flexible trimmer for Illumina sequence data. Bioinformatics.

[7] Li W., Godzik A. (2006). Cd-hit: a fast program for clustering and comparing large sets of protein or nucleotide sequences. Bioinformatics.

